# Remote Ischemic Preconditioning and Diazoxide Protect from Hepatic Ischemic Reperfusion Injury by Inhibiting HMGB1-Induced TLR4/MyD88/NF-κB Signaling

**DOI:** 10.3390/ijms20235899

**Published:** 2019-11-24

**Authors:** Won Uk Koh, Jiye Kim, Jooyoung Lee, Gi-Won Song, Gyu Sam Hwang, Eunyoung Tak, Jun-Gol Song

**Affiliations:** 1 Department of Anesthesiology, Asan Medical Center, University of Ulsan College of Medicine, 88 Olympic-ro 43-gil, Songpa-gu, Seoul 05505, Korea; koh9726@naver.com (W.U.K.); kshwang@amc.seoul.kr (G.S.H.); 2 Asan Institute for Life Sciences and Asan-Minnesota Institute for Innovating Transplantation, Asan Medical Center, University of Ulsan College of Medicine, 88 Olympic-ro 43-gil, Songpa-gu, Seoul 05505, Korea; gilkja@naver.com (J.K.); leejeyre@naver.com (J.L.); 3 Division of Liver Transplantation and Hepatobiliary Surgery, Asan-Minnesota Institute for Innovating Transplantation, Department of Surgery, Asan Medical Center, University of Ulsan College of Medicine, 88 Olympic-ro 43-gil, Songpa-gu, Seoul 05505, Korea; drsong71@amc.seoul.kr

**Keywords:** hepatic ischemia, ischemic preconditioning, ischemia-reperfusion, potassium channel

## Abstract

Remote ischemic preconditioning (RIPC) is known to have a protective effect against hepatic ischemia-reperfusion (IR) injury in animal models. However, the underlying mechanism of action is not clearly understood. This study examined the effectiveness of RIPC in a mouse model of hepatic IR and aimed to clarify the mechanism and relationship of the ATP-sensitive potassium channel (K_ATP_) and HMGB1-induced TLR4/MyD88/NF-κB signaling. C57BL/6 male mice were separated into six groups: (i) sham-operated control, (ii) IR, (iii) RIPC+IR, (iv) RIPC+IR+glyburide (K_ATP_ blocker), (v) RIPC+IR+diazoxide (K_ATP_ opener), and (vi) RIPC+IR+diazoxide+glyburide groups. Histological changes, including hepatic ischemia injury, were assessed. The levels of circulating liver enzymes and inflammatory cytokines were measured. Levels of apoptotic proteins, proinflammatory factors (TLR4, HMGB1, MyD88, and NF-κB), and IκBα were measured by Western blot and mRNA levels of proinflammatory cytokine factors were determined by RT-PCR. RIPC significantly decreased hepatic ischemic injury, inflammatory cytokine levels, and liver enzymes compared to the corresponding values observed in the IR mouse model. The K_ATP_ opener diazoxide + RIPC significantly reduced hepatic IR injury demonstrating an additive effect on protection against hepatic IR injury. The protective effect appeared to be related to the opening of K_ATP_, which inhibited HMGB1-induced TRL4/MyD88/NF-kB signaling.

## 1. Introduction

The induction of ischemia by clamping blood vessels of the target organ is frequently applied during surgical procedures to prevent blood loss and to secure an adequate surgical field. However, ischemia induced during surgery may also injure the target organ upon reperfusion due to activation of the immune response, oxidative stress, and inflammatory processes [1,2]. Hepatic ischemia-reperfusion (IR) injury can be caused by direct interventions to organs, including surgery, or by hypoxic damage to the liver [3,4]. This may result in hepatocyte and sinusoidal cell injury and perhaps necrosis or apoptosis, which can eventually lead to hepatic failure [1,5]. Several preconditioning strategies that have been proposed to reduce IR injury include direct transient clamping of the blood vessel of the target organ before ischemia [6,7] and remote ischemic preconditioning (RIPC) of distal limbs [8,9]. The protective effect of RIPC has been studied in several animal models, especially for heart and vascular surgeries [10,11,12].

The ATP-sensitive potassium channel (K_ATP_) is a member of the ATP-binding superfamily, which is located on different organs of the human body. It has been shown that RIPC protects the myocardium and brain from ischemic injury via opening of K_ATP_ channels [13]. However, little is known about the mechanism by which RIPC protects the liver against IR injuries and the involvement of K_ATP_ channels in this protective mechanism. The Toll-like receptor 4 (TLR4) is known to be responsible for the activation of proinflammatory signaling cascades in liver IR injury [14]. Thus, the relationship between the hepatic protective effect of RIPC and TLR4 signaling pathway also needs to be elucidated.

In this study, we investigated the effectiveness of RIPC in a mouse hepatic IR model and tried to elucidate the mechanisms of action and the role of K_ATP_ channel and high mobility group box 1 (HMGB1)-induced TLR4/myeloid differentiation primary response 88 (MyD88)/nuclear factor-kappa B (NF-κB) signaling.

## 2. Results

### 2.1. RIPC Decreases Hepatic IR Injury by Inhibition of the TLR4/MyD88/NF-κB Signaling

**Figure 1:**
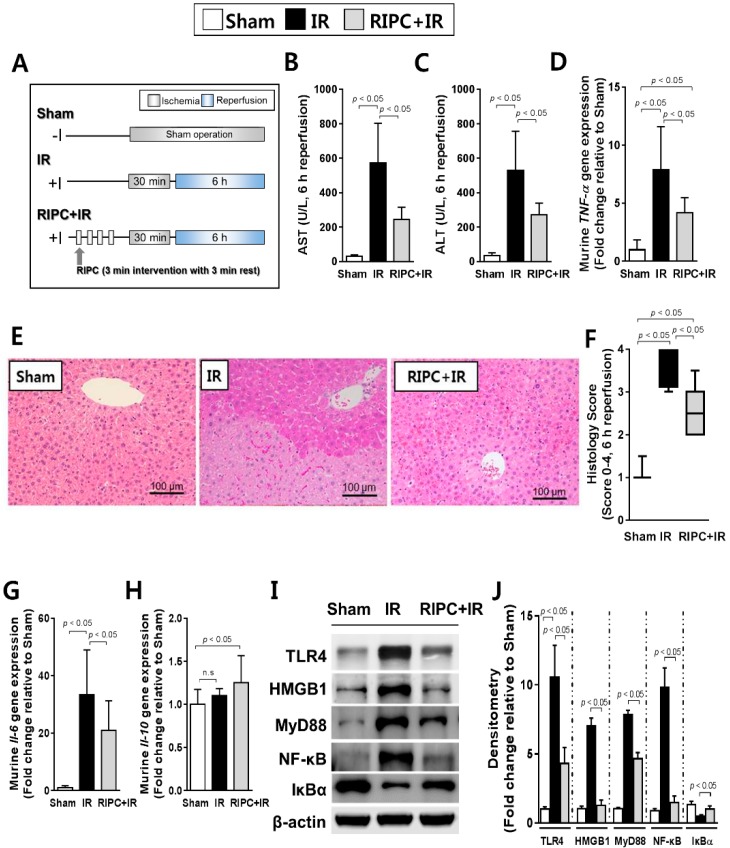
Characterization of remote ischemic preconditioning (RIPC)-treated ischemia-reperfusion (IR) mice. (**A**) study design; (**B**) aspartate aminotransferase (AST) and (**C**) alanine aminotransferase (ALT) serum levels; (**D**) Murine tumor necrosis factor-alpha (*TNF-α*) transcript levels as measured by RT-PCR. The housekeeping gene glyceraldehyde 3-phosphate dehydrogenase (*Gapdh)* served as an internal control; (**E**) hematoxylin and eosin staining of liver tissue samples; (**F**) Suzuki scoring index; 0-4. Data are presented as median and 10 to 90 percentile range; (**G**) murine hepatic interleukin-6 (*Il-6*) RT-PCR results. *Gapdh* again served as an internal control; (**H**) transcriptional levels of murine *Il-10*; (**I**) immunoblotting and (**J**) densitometry of nuclear factor-kappa B (NF-κB) pathway proteins (*n* = 10). β-actin was used as a loading control for all target proteins after stripping from the same membrane. Data are presented as mean ± standard deviation (SD) for 10 samples per group. *p* < 0.05, by one-way analysis of variance (ANOVA) followed by Bonferroni’s multiple comparisons tests were considered as significant.

When sham control, 30 min ischemia- and 6 h IR injury-induced, and RIPC-treated 30 min ischemia- and 6 h IR injury-induced mice (Figure 1A) were compared, the levels of aspartate aminotransferase (AST) and alanine aminotransferase (ALT) were significantly decreased in RIPC-treated IR mice (AST, 245.9 ± 68.9; ALT, 270.6 ± 68.6) compared to levels in IR only mice (AST, 573.2 ± 229.7; ALT, 530.0 ± 226.7; *p* < 0.05; Figure 1B,C). Reverse transcription polymerase chain reaction (RT-PCR) results revealed lower tumor necrosis factor-alpha (TNF-α) expression in the RIPC-treated IR mice (4.2 ± 1.3) than levels in IR mice (7.9 ± 3.7; *p* < 0.05; Figure 1D). In addition, there was markedly less liver tissue injury in sections from RIPC-treated IR mice (2.5, 2.0–3.5) than from IR mice (3.5, 3.0–4.0; Figure 1E); liver injury was quantified from 0–4 using the Suzuki histological scoring index (*p* < 0.05; Figure 1F). Hepatic interleukin (IL)-6 transcript levels were notably decreased in RIPC-treated IR mice (21.1 ± 10.2) compared to those in IR mice (33.4 ± 15.6; *p* < 0.05; Figure 1G). Hepatic *Il-10* transcript levels did not demonstrate difference between RIPC-treated IR mice (1.3 ± 0.3) compared to those in IR mice (1.1 ± 0.1; Figure 1H). To confirm the disruption of TLR4 signaling by RIPC, the protein levels of TLR4, HMGB1, MyD88, and NF-κB were measured; all were downregulated in RIPC-treated IR mice (TLR4, 4.3 ± 1.2; HMGB1, 1.3 ± 1.0; MyD88, 4.7 ± 0.8; NF-κB, 1.0 ± 0.6) compared to levels in IR mice (TLR4, 10.6 ± 2.3; HMGB1, 7.0 ± 1.2; MyD88, 7.9 ± 0.7; NF-κB, 9.8 ± 2.8; *p* < 0.05; Figure 1I,J).

### 2.2. The Effect of RIPC on Hepatic IR Injury Is Attenuated by K_ATP_ Blocker (Glyburide)

**Figure 2:**
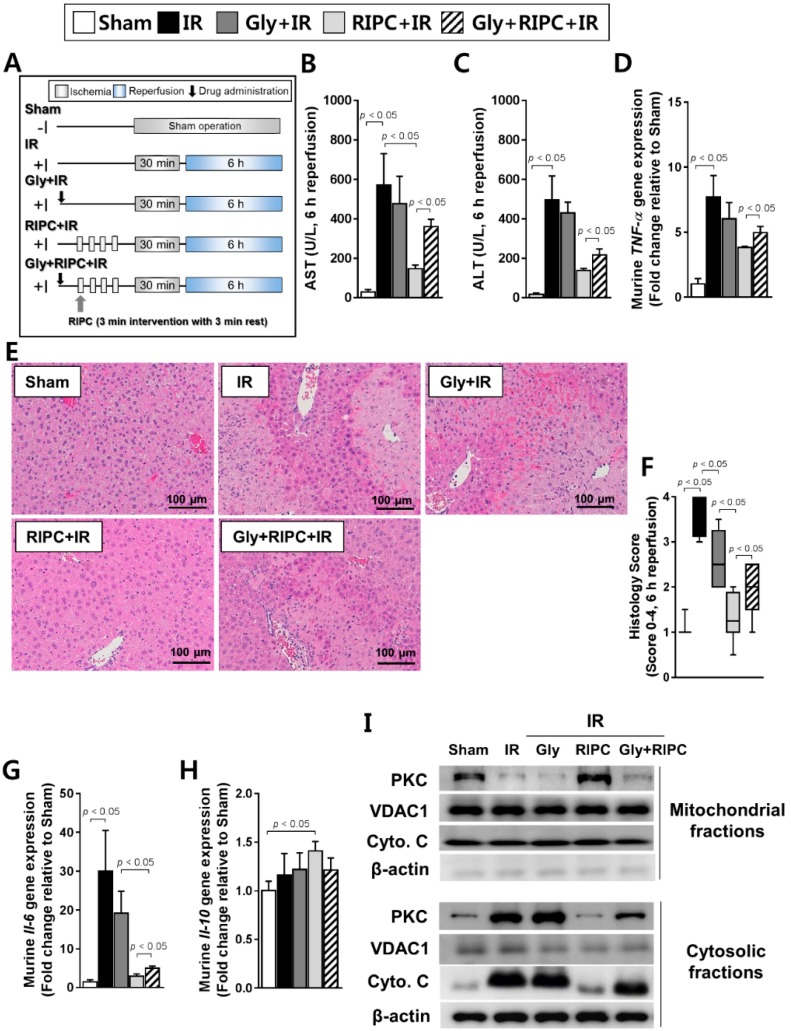
Effect of glyburide and RIPC treatment on IR mice. (**A**) animal experiment design; hepatic serum levels of (**B**) AST and (**C**) ALT; (**D**) levels of murine *TNF-α* as measured by RT-PCR; (**E**) hematoxylin and eosin staining of liver tissue; (**F**) histology scoring. Data are presented as median and 10 to 90 percentile range; (**G**) murine *Il-6* and (**H**) murine *Il-10* transcript levels; (**I**) immunoblotting of mitochondrial markers in mitochondrial and cytosolic lysates. β-actin was used as a loading control for all target proteins after stripping from the same membrane. Data are presented as mean ± SD for 10 samples per group. *p* < 0.05 by one-way ANOVA followed by Bonferroni’s multiple comparisons tests were considered as significant.

When the RIPC-treated IR mice were treated with the K_ATP_ blocker glyburide prior to RIPC (Figure 2A), the levels of the AST and ALT hepatic enzymes were significantly higher in the RIPC-treated IR group administered glyburide (AST, 360.0 ± 84.0; ALT, 216.3 ± 30.6) than in the absence of glyburide (AST, 146.5 ± 44.7; ALT, 138.3 ± 9.9; *p* < 0.05; Figure 2B,C). This effect was also observed in *TNF-α* mRNA levels (5.0 ± 1.3; 3.8 ± 0.2; *p* < 0.05; Figure 2D). In addition, there was a marked increase in necrosis and evident histological injury in sections from RIPC-treated IR mice administered glyburide (2.0, 1.0–2.5) compared to levels in animals not administered glyburide (1.3, 0.5–2.0; Figure 2E,F), where liver injury was quantified (*p* < 0.05; Figure 2F). Hepatic *Il-6* transcript levels were notably increased in RIPC-treated IR mice administered glyburide (5.0 ± 2.1) compared to those of RIPC-treated IR mice (2.9 ± 1.5; *p* < 0.05; Figure 2G). Hepatic *Il-10* transcript levels did not demonstrate a difference between RIPC-treated IR mice administered glyburide (1.2 ± 0.1) compared to those in RIPC-treated IR mice not administered glyburide (1.4 ± 0.1; Figure 2H). To perform further mechanistic studies with glyburide, we assessed whether glyburide induced mitochondrial damage was based on protein kinase C (PKC) immunoblotting. As shown in Figure 2I, PKC levels were lower in RIPC-treated IR mice administered glyburide than in sham control and RIPC-treated IR mice in mitochondrial fractions. In the cytosolic fraction, RIPC-treated IR mice administered glyburide had higher expression levels of PKC and cytochrome C than those in RIPC-treated IR mice (Figure 2I). Voltage-dependent anion-selective channel 1 (VDAC1) can be used as a mitochondrial marker, and cytochrome C is emitted from the mitochondria into the cytosol upon the initiation of apoptotic signaling. Using these parameters, we found that RIPC-treated IR mice administered glyburide acted through PKC and mitochondrial K_ATP_ in the hepatic IR mouse model.

### 2.3. Glyburide Decreases the Effect of RIPC by Attenuation of the TLR4/MyD88/NF-κB Signaling

**Figure 3:**
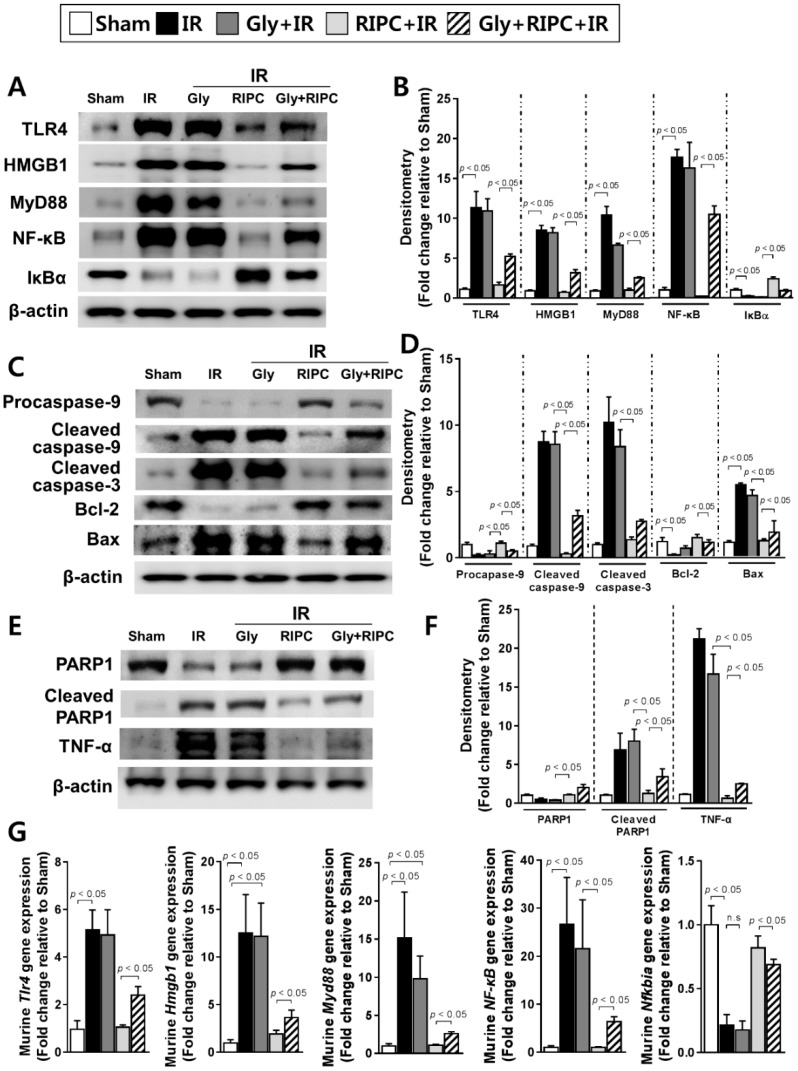
Immunoblotting of NF-κB signaling pathway molecules. (**A**) immunoblotting and (**B**) quantification of NF-κB signaling pathway protein expression levels; (**C**) immunoblotting and (**D**) densitometry of apoptosis-associated proteins; (**E**) immunoblotting and (**F**) quantification of total PARP1, cleaved PARP1, and TNF-α expression levels; (**G**) levels of NF-κB signaling pathway molecule transcripts. β-actin was used as a loading control for all target proteins after stripping from the same membrane. Data are presented as mean ± SD for 10 samples per group. *p* < 0.05 by one-way ANOVA followed by Bonferroni’s multiple comparisons tests were considered as significant.

To confirm the mechanisms underlying glyburide and RIPC treatment in the IR mouse model, immunoblotting of TLR4, HMGB1, MyD88, and NF-κB was performed. Levels of these proteins were notably higher in RIPC-treated IR mice administered glyburide (TLR4, 5.2 ± 0.7; HMGB1, 3.2 ± 0.9; MyD88, 2.5 ± 0.4; NF-κB, 10.4 ± 1.1) than levels in the absence of glyburide (TLR4, 1.6 ± 1.0; HMGB1, 0.7 ± 0.3; MyD88, 1.0 ± 0.4; NF-κB, 0.2 ± 0.0; *p* < 0.05; Figure 3A,B). The protein and mRNA level of IκBα was downregulated in RIPC-treated IR mice administered glyburide (0.9 ± 0.1 for protein; 0.7 ± 0.1 for mRNA) compared to that in the sham control and RIPC-treated IR mice without glyburide (2.4 ± 0.2 for protein; 0.8 ± 0.3 for mRNA; *p* < 0.05; Figure 3A,B,G). The levels of cleaved caspase-9, cleaved poly (ADP-ribose) polymerase 1 (PARP1), and the Bax/Bcl-2 ratio were higher in RIPC-treated IR mice administered glyburide (cleaved caspase-9, 3.2 ± 0.4; cleaved PARP1, 3.4 ± 1.0; Bax, 1.9 ± 0.9; Bcl-2, 1.1 ± 0.2) than those in RIPC-treated IR mice (cleaved caspase-9, 0.3 ± 0.1; cleaved PARP1, 1.2 ± 0.4; Bax, 1.2 ± 0.1; Bcl-2, 1.5 ± 0.2; *p* < 0.05; Figure 3C–F). In addition, levels of mRNAs encoding NF-κB pathway members were increased in RIPC-treated IR mice administered glyburide (*Tlr4*, 2.4 ± 0.8; *Hmgb1*, 3.7 ± 1.9; *Myd88*, 2.6 ± 0.8; *NF-κB*, 6.4 ± 2.7) compared to levels in RIPC-treated IR mice (*Tlr4*, 1.1 ± 0.3; *Hmgb1*, 1.9 ± 1.4; *Myd88*, 1.1 ± 0.3; *NF-κB*, 1.0 ± 0.4; *p* < 0.05; Figure 3G).

### 2.4. Additive Effects of K_ATP_ Agonist (Diazoxide) and RIPC on Acute Hepatic IR Injury

**Figure 4:**
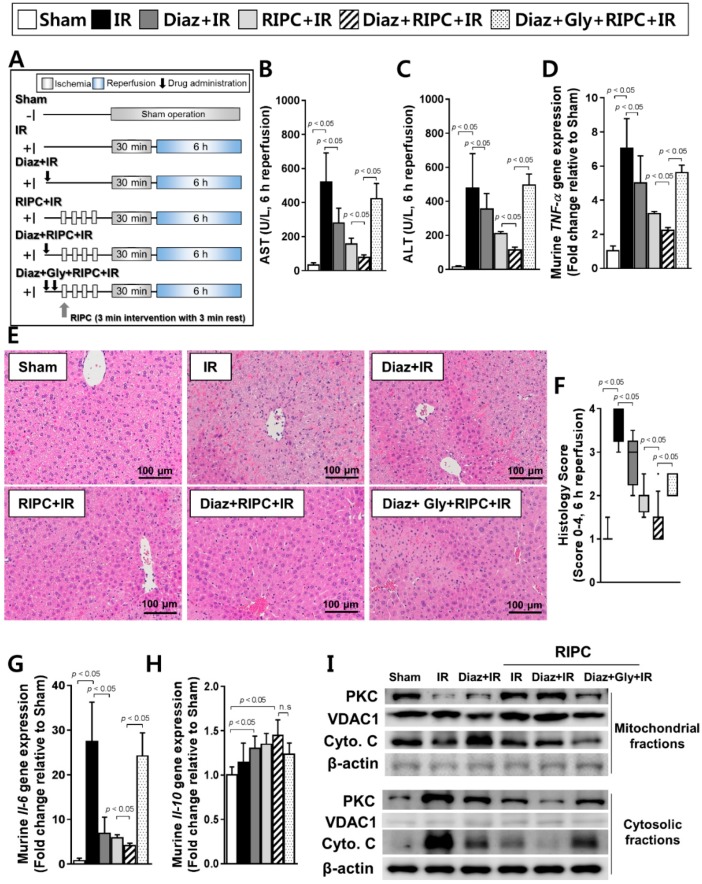
Characterization of diazoxide and RIPC-treated IR mice. (**A**) animal experiment design, murine hepatic serum levels of (**B**) AST and (**C**) ALT; (**D**) murine *TNF-α* transcript levels as measured by RT-PCR; (**E**) hepatic tissue histology; (**F**) histological scoring. Data are presented as median and 10 to 90 percentile range; transcriptional levels of murine (**G**) *Il-6* and (**H**) *Il-10*; (**I**) immunoblotting of mitochondrial proteins. β-actin was used as a loading control for all target proteins after stripping from the same membrane. Data are presented as mean ± SD for 10 samples per group. *p* < 0.05 by one-way ANOVA followed by Bonferroni’s multiple comparisons tests were considered as significant.

RIPC-treated IR mice also treated with diazoxide had lower AST (78.3 ± 53.7) and ALT (114.4 ± 49.8) levels compared to those of RIPC-treated IR mice without diazoxide (AST, 156.6 ± 117.0; ALT, 210.0 ± 29.7; *p* < 0.05; Figure 4B,C). RT-PCR revealed that the RIPC-treated IR mice administered diazoxide had significantly lower *TNF-α* mRNA levels (2.2 ± 0.4) than those of IR mice treated only with RIPC (3.2 ± 0.3; *p* < 0.05; Figure 4D). Histology examination also revealed that administering diazoxide to RIPC-treated IR mice resulted in significantly less injury (1.5, 1.0–2.0) than that in RIPC-treated IR mice (2.0, 1.5–2.5, Figure 4E) when liver injury was quantified (*p* < 0.05; Figure 4F). The transcript levels of *Il-6* were also markedly lower in RIPC-treated IR mice administered diazoxide (4.1 ± 0.6) than those in RIPC-treated IR mice (5.9 ± 0.7; *p* < 0.05; Figure 4G). Hepatic *Il-10* transcript levels did not demonstrate a difference between RIPC-treated IR mice administered diazoxide (1.4 ± 0.2) compared to those in RIPC-treated IR mice not administered diazoxide (1.3 ± 0.1; Figure 4H). When administered before inducing IR, diazoxide significantly reduced the markers of IR-induced hepatic injury (Figure 4B–G), and the synergic effect of diazoxide and RIPC was diminished when glyburide was administered alongside diazoxide prior to RIPC (Figure 4B–G). RIPC-treated IR mice administered diazoxide also had higher expression levels of PKC and apoptotic molecules, such as cytochrome C in mitochondrial fractions, and lower expression levels in cytosolic fractions than those of RIPC-treated IR mice (Figure 4I).

### 2.5. Diazoxide Prevents Hepatic Injury by Attenuating the TLR4/MyD88/NF-κB Pathway

**Figure 5:**
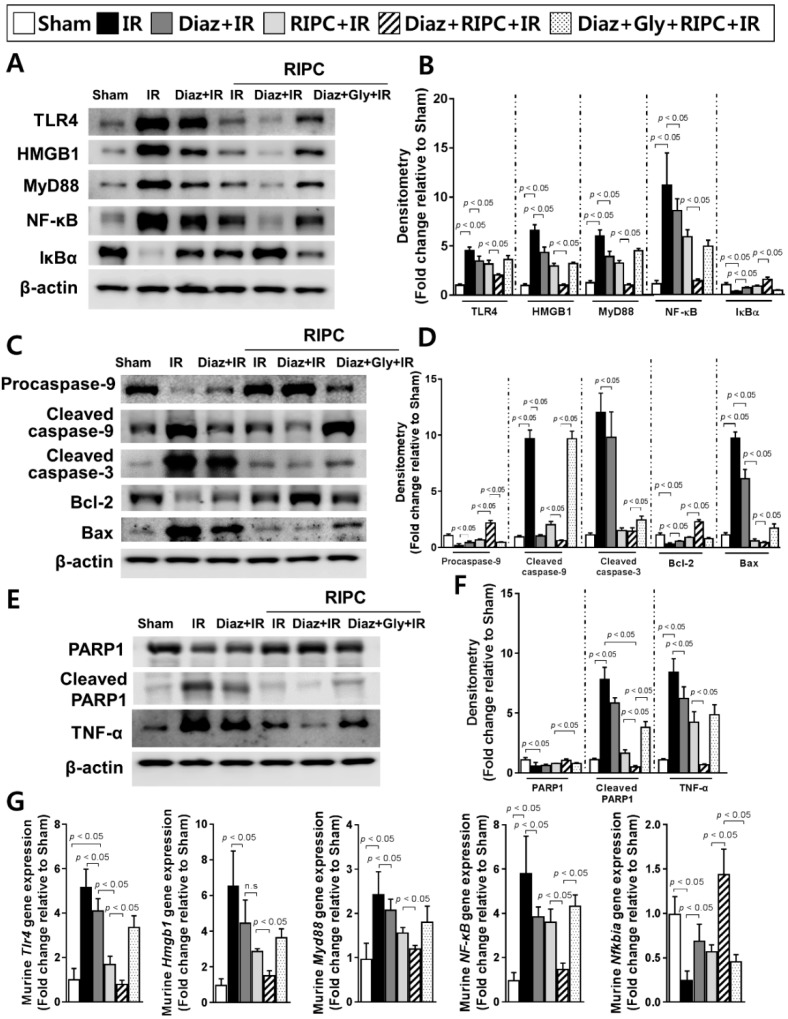
Effect of diazoxide and RIPC on signaling in IR mice. The effect of diazoxide and RIPC on signaling molecules associated with the NF-κB pathway was assessed. (**A**) Immunoblotting and (**B**) densitometry of NF-κB pathway molecules; (**C**) immunoblotting and (**D**) quantification of apoptosis-associated proteins; (**E**) immunoblotting and (**F**) densitometry of total PARP1, cleaved PARP1, and TNF-α; (**G**) fold-change in NF-κB signaling pathway molecule levels. β-actin was used as a loading control for all target proteins after stripping from the same membrane. Data are presented as mean ± SD for 10 samples per group. *p* < 0.05 by one-way ANOVA followed by Bonferroni’s multiple comparison tests were considered as significant.

To confirm the disruption of TLR4 signaling by RIPC and diazoxide, the expression levels of TLR4, HMGB1, MyD88, and NF-κB were measured. All were downregulated in RIPC-treated IR mice administered diazoxide (TLR4, 2.0 ± 0.1; HMGB1, 1.0 ± 0.1; MyD88, 1.0 ± 0.1; NF-κB, 1.4 ± 0.2) compared to levels in RIPC-treated IR mice (TLR4, 3.2 ± 0.4; HMGB1, 3.0 ± 0.3; MyD88, 3.3 ± 0.2; NF-κB, 5.9 ± 0.7; *p* < 0.05; Figure 5A,B). By contrast, IκBα, an inhibitor of NF-κB signaling activation, was upregulated in RIPC-treated IR mice administered diazoxide (1.6 ± 0.2; RIPC-treated IR mice, 0.9 ± 0.1; *p* < 0.05; Figure 5A,B,G). Next, the expression of apoptosis-associated proteins was assessed. The levels of cleaved caspase-9, cleaved PARP1, and the Bax/Bcl-2 ratio were lower in RIPC-treated IR mice administered diazoxide (cleaved caspase-9, 0.6 ± 0.1; cleaved PARP1, 0.5 ± 0.1; Bax, 0.4 ± 0.1; Bcl-2, 2.1 ± 0.2) than those in RIPC-treated IR mice (cleaved caspase-9, 2.0 ± 0.3; cleaved PARP1, 1.7 ± 0.3; Bax, 0.5 ± 0.2; Bcl-2, 0.8 ± 0.0; *p* < 0.05; Figure 5C–F). The levels of mRNAs encoding NF-κB pathway members were all decreased in RIPC-treated IR mice administered diazoxide (*Tlr4*, 0.8 ± 0.2; *Hmgb1*, 1.5 ± 0.3; *Myd88*, 1.2 ± 0.1; *NF-κ*B, 1.5 ± 0.3) compared to levels in RIPC-treated IR mice (*Tlr4*, 1.7 ± 0.4; *Hmgb1*, 2.8 ± 0.2; *Myd88*, 1.6 ± 0.1; *NF-κB*, 3.6 ± 0.6; *p* < 0.05; Figure 5G). The decreased expression levels of TLR4 signaling pathway-related proteins upon exposure to diazoxide with RIPC were attenuated by the addition of glyburide, demonstrating an increase in the levels of these proteins and apoptosis-associated proteins (Figure 5A–G).

## 3. Discussion

The present data indicate that RIPC before hepatic ischemia diminishes IR injury. This anti-hepatic ischemia effect was achieved via activating mitochondrial K_ATP_ (mK_ATP_) and inhibiting HMGB1-induced TLR4/MyD88/NF-κB signaling, and the combination of diazoxide and RIPC had an additive protective effect in this mouse model of IR. Circulating liver injury enzyme levels were lower and there was less histological evidence of liver injury after IR following RIPC or RIPC with diazoxide treatment compared to the observations in untreated IR mice. Furthermore, the hepatic expression of inflammatory cytokines and apoptosis-associated genes after IR injury was significantly lower in RIPC-treated IR mice administered diazoxide than those in RIPC-treated IR mice.

The preventive effectiveness of RIPC has been studied in various organs in several laboratory animal studies. RIPC can attenuate IR injury of the heart, liver, kidney, gastrointestinal organs, and the central nervous system, and can blunt the transmission of pain. The mechanism underlying the protective effect of RIPC has been intensively studied but remains unclear. Previous findings support an important role of mK_ATP_ in the protection from myocardial injury following IR, with the preventive effectiveness of RIPC for myocardial injury relying on K_ATP_ activation [15,16,17,18]. The activation of mK_ATP_ induces K^+^ uptake, which results in the activation of PKC and inhibits membrane permeability transition. The latter inhibition reduces calcium ion levels during ischemia and maintains mitochondrial integrity during reperfusion injury [19,20]. The involvement of mK_ATP_ in hepatic IR is also important. The preventive effectiveness of diazoxide against IR injury was previously described in a mouse liver transplant model [21]. This study demonstrated that diazoxide treatment markedly decreased the generation of apoptotic cells and tissue necrosis following IR injury of the liver, suggesting the involvement of mK_ATP_ as a possible mechanism. Our study results were consistent with the results of previous studies, demonstrating that diazoxide itself has protective effects against hepatic IR injury. Other in vivo and in vitro studies support the activation of mK_ATP_ as a key mechanism underlying protection from hepatic injury following IR [22,23,24,25,26]. Previous studies using the mouse model of liver transplant [21], and rat model of liver transplant and portal vein occlusion [22,23], have demonstrated that the activation of mK_ATP_ has a protective effect against ischemic liver damage and enhances liver regeneration following liver injury. However, the function of K_ATP_ in the preventive effectiveness of RIPC against liver IR injury is not currently well-characterized. The results of the present study further demonstrate that the protective effects of RIPC against liver IR are amplified by treatment with the K_ATP_ activator diazoxide, and, inversely, that the protective effects are dampened by treatment with the K_ATP_ inhibitor glyburide. The liver mitochondrial PKC fractions of the study animals were preserved after IR injury when RIPC or diazoxide was applied, but diminished when glyburide was administered before RIPC. These results suggest that the activation or opening of hepatic mitochondrial K_ATP_ channels is a key mechanism underlying the protective effect of RIPC against hepatic IR injury, supporting previous study results [22,23,24,25,26]. Furthermore, these protective effects of RIPC against liver IR injury appear to be mediated by K_ATP_ through inhibiting HMGB1-induced TLR4/MyD88/NF-κB signaling. Previous studies have demonstrated that the cardioprotective effect of RIPC against myocardial IR injury is mediated by inhibition of TLR4/MyD88/NF-κB signaling [27,28], but the involvement of this signaling pathway in liver IR injury is currently not well understood.

The inflammatory cytokine levels of TNF-α and IL-6 increase during liver IR injury. These cytokines undergo various interactions with other inflammatory cytokines and chemokines and activate products of reactive oxygen species (ROS), thereby initiating inflammatory processes. The elevated release of TNF-α also occurs during hepatic IR and, as confirmed by several previous studies, plays an important role in hepatocyte injury [29,30]. TNF-α release induces the apoptosis signaling cascade in hepatocytes, leading to programmed cell death. In the current study, RIPC and diazoxide administration decreased TNF-α and IL-6 levels, suggesting a protective effect against IR-induced hepatic inflammation, thereby preventing cell apoptosis or necroptosis. IR injury of the liver significantly increased caspase-3, caspase-9, and PARP1 expression, suggesting the apoptosis of hepatocytes. RIPC suppressed the expression of these enzymes after reperfusion, suggesting that RIPC defends against hepatic injury from IR by suppressing the cytochrome C, caspase-9, and caspase-3 release cascade through the activation of mK_ATP_. Importantly, this potential anti-hepatic ischemia effect was mediated through inhibition of HMGB1-induced TLR4/MyD88/NF-κB signaling. The involvement of inward rectifying potassium channels in the TLR4 signaling pathway has been previously demonstrated [31] and K_ATP_ opening protects cardiac cells by inhibiting the ROS-TLR4 pathway [32].

The role of the proinflammatory cytokine HMGB1 has been studied with much interest in various organ injuries and sepsis. This non-histone nuclear protein can activate inflammatory pathways by binding to TLR4, which leads to the subsequent binding of the adapter protein MyD88 and upregulation of NF-κB, causing an increase in inflammatory processes [33,34]. This HMGB1-TLR4 pathway plays a key role in the initiation phase of myocardial and acute lung IR injury, and also in IR-associated hepatic necrosis [35,36,37]. Recently, HMGB1 has been recognized as a therapeutic treatment for hepatic failure. Therefore, many modalities targeting HMGB1 and HMGB1-activated pathways have been studied in an effort to reduce hepatic IR injuries [38]. This includes inhibition of TLR4 [39], the use of anti-HMGB1 antibodies [40], and downregulation of nuclear HMGB1 [41]. The present results confirmed that acute hepatic injury following IR also involves the activation of the HMGB1-mediated TLR4/MyD88/NF-κB cascade, with RIPC alleviating this injury by inhibiting this pathway through the activation of hepatic mK_ATP_.

Our present study had several limitations. The most notable limitation is that glyburide is a nonselective K_ATP_ antagonist. Although the agonist diazoxide was used in this study as an mK_ATP_ opener to confirm the involvement of mK_ATP_ following RIPC protection after acute hepatic IR injury, the treatment with a specific blocker of mK_ATP_ may have provided more precise information. The main function of ROS in acute hepatic IR is relatively well-understood [42,43,44] and HMGB1 release from hepatocytes following ischemic injury is promoted by TLR4-dependent ROS production [44]. Thus, future investigations are required to determine the mechanism underlying the protective effect of RIPC involving ROS and HMGB1 interactions. These studies may further strengthen the results of this study and provide information on additional therapeutic targets for the protection against and treatment of these hepatic IR injuries.

## 4. Materials and Methods

This study was reviewed and approved by the Institutional Animal Care and Use Committee (IACUC) of the Asan Institute for Life Sciences, Asan Medical Center (11 May 2016). The committee follows the guidelines of the Institute of Laboratory Animal Resources (ILAR; Authorization no. 2016-02-129).

### 4.1. Study Group

C57BL/6 male mice (18–20 g, SPF) obtained from the Central Animal Incorporation was used. Mice were separated into eight groups: (i) sham-operated control, (ii) IR, (iii) RIPC-treated IR, (iv) glyburide- (K_ATP_ blocker) and IR, (v) glyburide- (K_ATP_ blocker) and RIPC-treated IR, (vi) diazoxide- (K_ATP_ opener) and IR, (vii) diazoxide- (K_ATP_ opener) and RIPC-treated IR, and (viii) diazoxide-, glyburide-, and RIPC-treated IR groups (each, *n* = 10). In the sham control group, the mice received a sham operation without additional interventions. The IR group received 30 min of liver ischemia followed by 6 h of reperfusion. In the RIPC group, mice received RIPC prior to the induction of IR. For the diazoxide- and glyburide-treated groups, the mice were treated with diazoxide (10 mg/kg) and/or glyburide (10 mg/kg) 1 h before the establishment of IR, just before applying RIPC in the hind limbs. The details are presented in Figure 1, Figure 2, and Figure 4.

### 4.2. Induction of RIPC and Liver IR

Thirty minutes after initiating anesthesia (10 mg/kg xylazine and 30 mg/kg zoletil), rubber bands were applied to the hind limbs of the mice to induce RIPC (3 min intervention with 3 min rest for 4 cycles) followed by 30 min of liver ischemia and 6 h reperfusion. Afterwards, mice were anesthetized for the period of induced ischemia using supplemental anesthesia solutions (10 mg/kg xylazine and 30 mg/kg zoletil). At the last step of the experiment, liver tissue and blood samples were harvested. Mice in the sham control and IR groups were treated using the same procedure as that for the experimental animals, but without the application of RIPC on the hind limbs. None of the mice developed further tissue injury following the application of the rubber bands and the animals were euthanized using carbon dioxide after harvesting liver tissue.

### 4.3. Blood Sampling and Laboratory Tests

Whole blood samples were collected from the inferior vena cava of anesthetized mice. The collected blood was allowed to clot for 30 min at 24 °C and centrifuged at 2500× *g* for 20 min at 4 °C. To separate the serum from the clotted cells, the transparent upper phase was transferred into new tubes. Serum AST and ALT levels were detected using an automated analyzer (Hitachi 7180, Tokyo, Japan).

### 4.4. Isolation of mRNA and RT-PCR

The levels of pro-inflammatory cytokines TNF-α, IL-6, and anti-inflammatory cytokine IL-10 were measured. Additionally, to elucidate the mechanisms underlying the anti-inflammatory effectiveness of RIPC, diazoxide, and glyburide, the relationship between murine HMGB1-induced TLR4/MyD88/NF-κB signaling was investigated. TLR4 is an important activator of IR-induced inflammatory responses in the liver. Left lobes of hepatic samples were harvested. Tissue (10 mg) from each sample was used to isolate total RNA using a commercial kit (Macherey & Nagel, Dueren, Germany). Complementary DNA was synthesized by performing reverse transcription with an iScript complementary DNA Synthesis kit (Bio-Rad, Hercules, CA, USA). For PCR, the primer sets contained 1 μM each of the sense and antisense primers and SYBR Green (Molecular Probes, New Brunswick, NJ, USA). Murine *TNF-α* (Cat no. QT00104006) and *Il-6* (Cat no. QT0098875) commercial primers were purchased from Qiagen (Valencia, CA, USA). The sense and antisense primer sequences were shown in Table S1. *Gapdh* was used as a control. The mRNA levels and fold-change were measured as previously described [45].

### 4.5. Immunoblotting

The levels of cleaved caspase-9, cleaved caspase-3, total PARP1, cleaved PARP1, Bax, and Bcl-2 were also quantified. Hepatic proteins from the left lobe were separated by 15% polyacrylamide gel electrophoresis and transferred to a membrane for the next steps. Membranes were blocked with 5% nonfat dry milk (Applichem, Cheshire, CT, USA) in Tris-buffered saline-Tween-20 and then incubated with anti-HMGB1 (39 kDa; Santa Cruz Biotechnology, Dallas, TX, USA) followed by goat anti-rabbit horseradish peroxidase. Protein signals were detected by enhanced chemiluminescence (ECL). For the loading control, blots were washed and reprobed for actin using the HRP conjugated monoclonal anti-β-actin antibody (A3854; Sigma-Aldrich, St. Louis, MO, USA). The membrane was washed and the proteins were detected by ECL. The details of antibodies are shown in Table S2.

### 4.6. Hepatic Tissue Histology

The left liver lobes were harvested from the mice and fixed in 4.5% buffered formalin, dehydrated, and embedded in paraffin. Histology examination of sections to quantify hepatic injury was performed using hematoxylin and eosin staining as previously described [46].

### 4.7. Statistical Analyses

Data were analyzed using GraphPad Prism 5.0 software (GraphPad Software, La Jolla, CA, USA). Hepatic tissue injury scores are described as medians and ranges. All the data are described as mean ± standard deviation for 10 samples per condition. Immunoblotting data were obtained from two procedures. Statistical comparisons were performed using one-way analysis of variance (ANOVA) with Bonferroni’s correction. A *p*-value < 0.05 was considered statistically significant.5. Conclusions

In conclusion, extensive acute liver injury was induced using an in vivo mouse acute hepatic IR model. Applying RIPC before acute hepatic IR diminished the level of injury and action was amplified with the mK_ATP_ activator, diazoxide. This anti-hepatic ischemia effect was achieved via activating mK_ATP_ and inhibiting HMGB1-induced TLR4/MyD88/NF-κB signaling. Based on these study results, we observed the effectiveness of RIPC against acute liver IR injury and highlighted HMGB1-induced TLR4/MyD88/NF-κB signaling as an underlying therapeutic remedy for preventing IR injury in patients receiving major hepatectomy or liver transplantation.

## Supplementary Materials

Supplementary materials can be found at https://www.mdpi.com/1422-0067/20/23/5899/s1.

## References

[1] Klune J.R., Tsung A. (2010). Molecular biology of liver ischemia/reperfusion injury: Established mechanisms and recent advancements. Surg. Clin. N. Am..

[2] Carden D.L., Granger D.N. (2000). Pathophysiology of ischaemia-reperfusion injury. J. Pathol..

[3] Zhou H., Zhu J., Yue S., Lu L., Busuttil R.W., Kupiec-Weglinski J.W., Wang X., Zhai Y. (2016). The dichotomy of endoplasmic reticulum stress response in liver ischemia-reperfusion injury. Transplantation.

[4] Kimura K., Shirabe K., Yoshizumi T., Takeishi K., Itoh S., Harimoto N., Ikegami T., Uchiyama H., Okano S., Maehara Y. (2016). Ischemia-reperfusion injury in fatty liver is mediated by activated NADPH oxidase 2 in rats. Transplantation.

[5] Massip-Salcedo M., Rosello-Catafau J., Prieto J., Avila M.A., Peralta C. (2007). The response of the hepatocyte to ischemia. Liver Int..

[6] Bahde R., Spiegel H.U. (2010). Hepatic ischaemia-reperfusion injury from bench to bedside. Br. J. Surg..

[7] Lesurtel M., Lehmann K., de Rougemont O., Clavien P.A. (2009). Clamping techniques and protecting strategies in liver surgery. HPB (Oxford).

[8] Abu-Amara M., Yang S.Y., Quaglia A., Rowley P., Tapuria N., Seifalian A.M., Fuller B.J., Davidson B.R. (2011). Effect of remote ischemic preconditioning on liver ischemia/reperfusion injury using a new mouse model. Liver Transplant..

[9] Kanoria S., Jalan R., Davies N.A., Seifalian A.M., Williams R., Davidson B.R. (2006). Remote ischaemic preconditioning of the hind limb reduces experimental liver warm ischaemia-reperfusion injury. Br. J. Surg..

[10] Li D.Y., Shi X.J., Li W., Sun X.D., Wang G.Y. (2016). Ischemic preconditioning and remote ischemic preconditioning provide combined protective effect against ischemia/reperfusion injury. Life Sci..

[11] Oberkofler C.E., Limani P., Jang J.H., Rickenbacher A., Lehmann K., Raptis D.A., Ungethuem U., Tian Y., Grabliauskaite K., Humar R. (2014). Systemic protection through remote ischemic preconditioning is spread by platelet-dependent signaling in mice. Hepatology.

[12] Lai I.R., Chang K.J., Chen C.F., Tsai H.W. (2006). Transient limb ischemia induces remote preconditioning in liver among rats: The protective role of heme oxygenase-1. Transplantation.

[13] Hu X., Yang Z., Yang M., Qian J., Cahoon J., Xu J., Sun S., Tang W. (2014). Remote ischemic preconditioning mitigates myocardial and neurological dysfunction via K(ATP) channel activation in a rat model of hemorrhagic shock. J. Exp. Med..

[14] McDonald K.A., Huang H., Tohme S., Loughran P., Ferrero K., Billiar T., Tsung A. (2015). Toll-like receptor 4 (TLR4) antagonist eritoran tetrasodium attenuates liver ischemia and reperfusion injury through inhibition of high-mobility group box protein B1 (HMGB1) signaling. Mol. Med..

[15] Kevelaitis E., Oubenaissa A., Mouas C., Peynet J., Menasche P. (2000). Opening of mitochondrial potassium channels: A new target for graft preservation strategies?. Transplantation.

[16] Liu Y., Sato T., Seharaseyon J., Szewczyk A., O’Rourke B., Marban E. (1999). Mitochondrial ATP-dependent potassium channels. Viable candidate effectors of ischemic preconditioning. Ann. N. Y. Acad. Sci..

[17] Mabanta L., Valane P., Borne J., Frame M.D. (2006). Initiation of remote microvascular preconditioning requires K_ATP_ channel activity. Am. J. Physiol. Heart Circ. Physiol..

[18] Konstantinov I.E., Li J., Cheung M.M., Shimizu M., Stokoe J., Kharbanda R.K., Redington A.N. (2005). Remote ischemic preconditioning of the recipient reduces myocardial ischemia-reperfusion injury of the denervated donor heart via a Katp channel-dependent mechanism. Transplantation.

[19] Garlid K.D., Costa A.D., Quinlan C.L., Pierre S.V., Dos Santos P. (2009). Cardioprotective signaling to mitochondria. J. Mol. Cell. Cardiol..

[20] Garlid K.D., Paucek P., Yarov-Yarovoy V., Murray H.N., Darbenzio R.B., D’Alonzo A.J., Lodge N.J., Smith M.A., Grover G.J. (1997). Cardioprotective effect of diazoxide and its interaction with mitochondrial ATP-sensitive K+ channels. Possible mechanism of cardioprotection. Circ. Res..

[21] Wu Q., Tang C., Zhang Y.J., Jiang Y., Li X.W., Wang S.G., Bie P. (2011). Diazoxide suppresses hepatic ischemia/reperfusion injury after mouse liver transplantation by a BCL-2-dependent mechanism. J. Surg. Res..

[22] Nakagawa Y., Yoshioka M., Abe Y., Uchinami H., Ohba T., Ono K., Yamamoto Y. (2012). Enhancement of liver regeneration by adenosine triphosphate-sensitive K+ channel opener (diazoxide) after partial hepatectomy. Transplantation.

[23] Grossini E., Pollesello P., Bellofatto K., Sigaudo L., Farruggio S., Origlia V., Mombello C., Mary D.A., Valente G., Vacca G. (2014). Protective effects elicited by levosimendan against liver ischemia/reperfusion injury in anesthetized rats. Liver Transplant..

[24] Zeng Z., Huang H.F., He F., Wu L.X., Lin J., Chen M.Q. (2012). Diazoxide attenuates ischemia/reperfusion injury via upregulation of heme oxygenase-1 after liver transplantation in rats. World J. Gastroenterol..

[25] Nogueira M.A., Coelho A.M., Sampietre S.N., Patzina R.A., Pinheiro da Silva F., D’Albuquerque L.A., Machado M.C. (2014). Beneficial effects of adenosine triphosphate-sensitive K+ channel opener on liver ischemia/reperfusion injury. World J. Gastroenterol..

[26] Ibrahim M.A., Abdel-Gaber S.A., Amin E.F., Ibrahim S.A., Mohammed R.K., Abdelrahman A.M. (2014). Molecular mechanisms contributing to the protective effect of levosimendan in liver ischemia-reperfusion injury. Eur. J. Pharmacol..

[27] Zhang J., Zhang J., Yu P., Chen M., Peng Q., Wang Z., Dong N. (2017). Remote Ischaemic Preconditioning and Sevoflurane Postconditioning Synergistically Protect Rats from Myocardial Injury Induced by Ischemia and Reperfusion Partly via Inhibition TLR4/MyD88/NF-kappaB Signaling Pathway. Cell. Physiol. Biochem..

[28] Wang Y., Wang L., Li J.H., Zhao H.W., Zhang F.Z. (2019). Morphine alleviates myocardial ischemia/reperfusion injury in rats by inhibiting TLR4/NF-kappaB signaling pathway. Eur. Rev. Med. Pharmacol. Sci..

[29] Colletti L.M., Cortis A., Lukacs N., Kunkel S.L., Green M., Strieter R.M. (1998). Tumor necrosis factor up-regulates intercellular adhesion molecule 1, which is important in the neutrophil-dependent lung and liver injury associated with hepatic ischemia and reperfusion in the rat. Shock.

[30] Tang Z.Y., Loss G., Carmody I., Cohen A.J. (2006). TIMP-3 ameliorates hepatic ischemia/reperfusion injury through inhibition of tumor necrosis factor-alpha-converting enzyme activity in rats. Transplantation.

[31] Jo H.Y., Kim S.Y., Lee S., Jeong S., Kim S.J., Kang T.M., Lee K.Y. (2011). Kir3.1 channel is functionally involved in TLR4-mediated signaling. Biochem. Biophys. Res. Commun..

[32] Liang W., Chen M., Zheng D., Li J., Song M., Zhang W., Feng J., Lan J. (2017). The opening of ATP-sensitive K+ channels protects H9c2 cardiac cells against the high glucose-induced injury and inflammation by inhibiting the ROS-TLR4-necroptosis pathway. Cell. Physiol. Biochem..

[33] Park J.S., Gamboni-Robertson F., He Q., Svetkauskaite D., Kim J.Y., Strassheim D., Sohn J.W., Yamada S., Maruyama I., Banerjee A. (2006). High mobility group box 1 protein interacts with multiple Toll-like receptors. Am. J. Physiol. Cell Physiol..

[34] Hreggvidsdottir H.S., Lundberg A.M., Aveberger A.C., Klevenvall L., Andersson U., Harris H.E. (2012). High mobility group box protein 1 (HMGB1)-partner molecule complexes enhance cytokine production by signaling through the partner molecule receptor. Mol. Med..

[35] Abraham E., Arcaroli J., Carmody A., Wang H., Tracey K.J. (2000). HMG-1 as a mediator of acute lung inflammation. J. Immunol..

[36] Andrassy M., Volz H.C., Igwe J.C., Funke B., Eichberger S.N., Kaya Z., Buss S., Autschbach F., Pleger S.T., Lukic I.K. (2008). High-mobility group box-1 in ischemia-reperfusion injury of the heart. Circulation.

[37] Tsung A., Sahai R., Tanaka H., Nakao A., Fink M.P., Lotze M.T., Yang H., Li J., Tracey K.J., Geller D.A. (2005). The nuclear factor HMGB1 mediates hepatic injury after murine liver ischemia-reperfusion. J. Exp. Med..

[38] Yamamoto T., Tajima Y. (2017). HMGB1 is a promising therapeutic target for acute liver failure. Expert Rev. Gastroenterol. Hepatol..

[39] Yokoi T., Yokoyama Y., Kokuryo T., Yamaguchi J., Nagino M. (2017). Inhibition of Toll-like receptor 4 ameliorates experimental postischemic injury in the cholestatic liver through inhibition of high-mobility group box protein b1 (HMGB1) signaling. Surgery.

[40] Sugihara M., Sadamori H., Nishibori M., Sato Y., Tazawa H., Shinoura S., Umeda Y., Yoshida R., Nobuoka D., Utsumi M. (2016). Anti-high mobility group box 1 monoclonal antibody improves ischemia/reperfusion injury and mode of liver regeneration after partial hepatectomy. Am. J. Surg..

[41] Zhao G., Fu C., Wang L., Zhu L., Yan Y., Xiang Y., Zheng F., Gong F., Chen S., Chen G. (2017). Down-regulation of nuclear HMGB1 reduces ischemia-induced HMGB1 translocation and release and protects against liver ischemia-reperfusion injury. Sci. Rep..

[42] Zhang W., Wang M., Xie H.Y., Zhou L., Meng X.Q., Shi J., Zheng S. (2007). Role of reactive oxygen species in mediating hepatic ischemia-reperfusion injury and its therapeutic applications in liver transplantation. Transplant. Proc..

[43] Tejima K., Arai M., Ikeda H., Tomiya T., Yanase M., Inoue Y., Nagashima K., Nishikawa T., Watanabe N., Omata M. (2004). Ischemic preconditioning protects hepatocytes via reactive oxygen species derived from Kupffer cells in rats. Gastroenterology.

[44] Tsung A., Klune J.R., Zhang X., Jeyabalan G., Cao Z., Peng X., Stolz D.B., Geller D.A., Rosengart M.R., Billiar T.R. (2007). HMGB1 release induced by liver ischemia involves Toll-like receptor 4 dependent reactive oxygen species production and calcium-mediated signaling. J. Exp. Med..

[45] Eltzschig H.K., Ibla J.C., Furuta G.T., Leonard M.O., Jacobson K.A., Enjyoji K., Robson S.C., Colgan S.P. (2003). Coordinated adenine nucleotide phosphohydrolysis and nucleoside signaling in posthypoxic endothelium: Role of ectonucleotidases and adenosine A2B receptors. J. Exp. Med..

[46] Hart M.L., Much C., Kohler D., Schittenhelm J., Gorzolla I.C., Stahl G.L., Eltzschig H.K. (2008). Use of a hanging-weight system for liver ischemic preconditioning in mice. Am. J. Physiol. Gastrointest. Liver Physiol..

