# Supplementary material for: Remote Ischemic Preconditioning and Diazoxide Protect from Hepatic Ischemic Reperfusion Injury by Inhibiting HMGB1-Induced TLR4/MyD88/NF-κB Signaling
Source: Int J Mol Sci. 2019 Nov 24;20(23):5899. doi: 10.3390/ijms20235899 (PMC6929132; doi:10.3390/ijms20235899)
Supplement: Supplementary file 1 [file ijms-20-05899-s001.pdf]

Supplementary table 1

Gene		5' – 3'
Il-6	Sequence not provided	QuantiTect Primer Assay Mm_Il6_1_SG (Cat. No. QT0098875; Qiagen)
TNF-α	Sequence not provided	QuantiTect Primer Assay Mm_Tnf_1_SG (Cat. No. QT00104006; Qiagen)
Hmgb1	sense	CCA AAG CGA TAG GAA ACT GC
	anti-sense	TTT TGC CTC TCG GCT TTT TA
Il-10	sense	CCA AGC CTT ATC GGA AAT GA
	anti-sense	TTT TCA CAG GGG AGA AAT CG
Nfkbia	sense	AGG ACG AGG AGT ACG AGC AA
	anti-sense	GTC TCC CTT CAC CTG ACC AA
Myd88	sense	ACT GGC CTG AGC AAC TAG GA
	anti-sense	CGT GCC ACT ACC TGT AGC AA
NF-κB	sense	TCA GAC ACC TCT GCA CTT GG
	anti-sense	GCA GGC TAT TGC TCA CA
Tlr4	sense	GGC AGC AGG TGG AAT TGT AT
	anti-sense	AGG CCC CAG AGT TTT GTT CT
Gapdh	sense	AGG TCG GTG TGA ACG GAT TTG
	anti-sense	TGT AGA CCA TGT AGT TGA GGT CA

Supplementary table 2

Antigen	Host	Dilution	Manufacture	Cat. no.
Bax	Mouse	1:1,000	Santa Cruz	sc-7480
Bcl-2	Mouse	1:1,000	Santa Cruz	sc-7382
Caspase-9	Mouse	1:1,000	Cell Signaling	9508S
Cleaved caspase-3	Rabbit	1:1,000	Cell Signaling	9664S
Cytochrome C	Rabbit	1:1,000	Abcam	ab133504
HMGB1	Rabbit	1:1,000	Abcam	ab79823
IκBα	Rabbit	1:1,000	Abcam	ab32518
MyD88	Rabbit	1:1,000	Abcam	ab2068
NF-κB	Rabbit	1:1,000	Abcam	ab32360
PARP1	Rabbit	1:1,000	Abcam	ab32138
PKC	Rabbit	1:1,000	Abcam	ab124806
TNF-α	Rabbit	1:1,000	Abcam	ab9635
TLR4	Rabbit	1:1,000	Abcam	ab22048
VDAC1	Mouse	1:1,000	Santa Cruz	sc-390996
β-actin	Mouse (HRP-conjugated)	1:20,000	Sigma-Aldrich	A3854
